# Supplementary material for: Genome mining unearths a hybrid nonribosomal peptide synthetase-like-pteridine synthase biosynthetic gene cluster
Source: eLife. 2017 Mar 15;6:e25229. doi: 10.7554/eLife.25229 (PMC5384830; doi:10.7554/eLife.25229)
Supplement: Supplementary file 1. — (A) Molecular feature list dependent on the presence of the wild-type pathway. (B-D) Primers used. DOI: http://dx.doi.org/10.7554/eLife.25229.035 [file elife-25229-supp1.zip › Suplementary file 1/Supplementary file 1B-D.docx]

**Supplementary file 1A**

Attached as Excel workbook.

**Molecular feature list dependent on the presence of the wild-type pathway.** Shown are the observed masses for wild-type dependent features detected in 5 out of 5 biological replicates after automated and manual removal of control features. If a feature was detected in any 1 of the 5 control replicates, it was defined as a control molecular feature. The average intensity for each one of the wild-type features is shown, as well as their average intensity in each deletion construct. An intensity of one (highlighted red) denotes the absence of feature detection in the given data set. The number in parentheses immediately to the right of the intensity value corresponds to the number of biological replicates in which the feature is found. Features dependent on a given enzyme are tabulated.

**Supplementary file 1B**

| Primer Name | Sequence |
| --- | --- |
| 2796-cluster-5 (NdeI) | 5’-gtaaaattcatatgccgacggtggagccaggaga |
| 2796-cluster-3 (XhoI) | 5’-gtaaaattctcgagctaatgaatgttagtagaaacagatcgaaatgtttg |
| Delplu2793F | 5’-ttgtttaactttaagaaggagatatacat**atgacaattcaatatattactcgcaaaggctc** |
| Delplu2793R | 5’-**tgcgagtaatatattgaattgtcat**atgtatatctccttcttaaagttaaacaaaattatttctagag |
| Delplu2794F | 5’-gaattgattagaggtattgcatga**agtaacgaatcacgatgaatgagggttg** |
| Delplu2794R | 5’-**ctcattcatcgtgattcgttact**tcatgcaatacctctaatcaattcatagaactc |
| Delplu2795F | 5’-gatgaatgagggttgacgaat**atgtctttaattgaaattaatacgaccacaatagg** |
| Delplu2795R | 5’-**gtcgtattaatttcaattaaagacat**attcgtcaaccctcattcatcgtg |
| Delplu2796F | 5’-aaattaatacgaccacaataggtatatt**tcaagcatgataccagctcaagaattacac** |
| Delplu2796R | 5’-**cttgagctggtatcatgcttga**aatatacctattgtggtcgtattaatttcaattaaagac |
| Delplu2797F | 5’-gtgaaaccattgagtaatcaagc**tttatctgtattcgtgagatctactttaatgacg** |
| Delplu2797R | 5’-**ttaaagtagatctcacgaatacagataaa**gcttgattactcaatggtttcacaagtatctttaag |
| Delplu2798F | 5’-gatctactttaatgacggaaaatcaat**aagttccacgctagcaacatacggtttc** |
| Delplu2798R | 5’-**tatgttgctagcgtggaactt**attgattttccgtcattaaagtagatctcacg |
| Delplu2799F | 5’-tttaattttttgcgagtaaatatgat**ctcgagcaccaccaccaccac** |
| Delplu2799R | 5’-**tggtggtggtgctcgag**atcatatttactcgcaaaaaattaaatcacctg |
| PluS434AF | 5’-caaactcggcggtgac**g**cgatgaaggctgcccacacgatatc |
| PluS434AR | 5’-gtgtgggcagccttcatcg**c**gtcaccgccgagtttgaagaaattg |

**Oligonucleotides (Integrated DNA Technologies (IDT), USA; or Keck Foundation Biotechnology Resource Laboratory at Yale University) used for biosynthetic pathway isolation and genetic modification.** Underlined sequences represent restriction digestion sites utilized in cloning. For genetic deletion primers, the bold-faced sequence portion represents that which is immediately downstream of the gene to be excised. Likewise, the normal text represents the upstream sequence. For point mutagenesis primers, the bold face nucleotide represents the modification.

**Supplementary file 1C**

| Primer Name | Sequence |
| --- | --- |
| Seq 1 | 5’-tcatacaagaacggttaacagcagcaat |
| Seq T7P*1 | 5’-cgatggcattgtattcgacggc |
| Seq 2 | 5’-agtactgtaatccaacagttgagaatattgcc |
| Seq 3 | 5’-acaggcgatgttacaggactgatcg |
| Seq 4 | 5’-gatgaaatcgaacactttaatactatcgatcc |
| Seq 5 | 5’-aaagttccattagtgatccggacacc |
| Seq 6 | 5’-actgaaattttgggtaagttattacctcgtaca |
| Seq 7 | 5’-atctcgaacaccacagaattaatccttca |
| Seq 8 | 5’-tatgtctgccgataccatgaaaacaaat |
| Seq 9 | 5’-aaaattcacccagaaaattacacagctg |
| Seq 10 | 5’-tcaactttctatgcccatgcatgc |
| Seq 11 | 5’-acaattgccgatgcactcgct |
| Seq 12 | 5’-ctactgtttatacatgacatttataatcaacaatgc |
| Seq 13 | 5’-caagtccatatcagtctaggagattatgaaacc |
| Seq 14 | 5’-aaatactggttatcggaagcttataatgtggta |
| Seq 15 | 5’-atattaatctaaaaataccgttttttctgatccaa |
| Seq 16 | 5’-ttagtcagccttattacaacttgttaacagca |

**Oligonucleotides (IDT or Keck Foundation Biotechnology Resource Laboratory at Yale University) used for primer walking and sequence validation of the pepteridine biosynthetic pathway and mutant constructs.**

**Supplementary file 1D**

| Primer Name | Sequence |
| --- | --- |
| Up-F (SacI) | 5’-ctgaagctgagctctcagataagctatcgccataaaagggacaac |
| Up-R | 5’- gataacaatgtgtcgtttttggcccttttgcaaaaatgtattcgttataattaattaaaga  agtaggtaacagtcg |
| Dwn-F | 5’-cgactgttacctacttctttaattaattataacgaatacatttttgcaaaagggccaaaaa  cgacacattgttatc |
| Dwn-R (SacI) | 5’-ctgaagctgagctcgtattgaatggaaagcggggtctgttg |
| pDS-F | 5’-gaacggcaggtatatgtgatggg |
| pDS-R | 5’-agcaattttgagtgacacaggaacac |
| CDSSeq-1 | 5’-cctttataattaacaagtaacgcaatgc |
| CDSSeq-2 | 5’-gatatttcccctcaactatttgtg |
| 99Seq-1 | 5’-caagtgatgcaaagttaacctcggtc |
| 99Seq-2 | 5’-cagagcacattcatcaccgatatttgc |
| LocUp | 5’-gaggatatggagaagcagaagcagc |
| LocDwn | 5’-acgttggatctgatccgtgaatatcg |

**Oligonucleotides (Keck Foundation Biotechnology Resource Laboratory at Yale University) used for construction and sequence validation of the allelic-exchange plasmid and pepteridine locus deletion constructs.** Underlined sequences represent restriction digestion sites utilized in cloning.
